# Repair of coronary artery ostium with a ring-shaped bovine pericardial patch

**DOI:** 10.1093/icvts/ivac251

**Published:** 2022-10-13

**Authors:** Dong Zhang, Gui-Jun Zhu, Xiang-Yang Wei, Bin Li, Jie Hu, Wei-Guo Ma, Xing-Peng Chen

**Affiliations:** Department of Cardiothoracic Surgery, Luoyang Central Hospital, Luoyang, China; Department of Cardiothoracic Surgery, Luoyang Central Hospital, Luoyang, China; Department of Cardiothoracic Surgery, Luoyang Central Hospital, Luoyang, China; Department of Cardiothoracic Surgery, Luoyang Central Hospital, Luoyang, China; Division of Cardiac Surgery, Department of Surgery, Yale University School of Medicine, New Haven, CT, USA; Department of Cardiothoracic Surgery, Luoyang Central Hospital, Luoyang, China

**Keywords:** Coronary artery/ostium, Aortic dissection, Endocarditis, Bovine pericardial patch, Surgical outcomes

## Abstract

As an approach to coronary artery ostial injury in type A aortic dissection and infective endocarditis, we describe a technique of coronary ostial repair using a ring-shaped bovine pericardial patch. The inner and outer rims of the patch were sutured to the involved coronary ostium (to close the ostial tear) and to the aortic wall (to cover the sinus), respectively. Four patients were successfully managed and computed tomographic coronary arteriogram at follow-up showed patent coronary ostia and arteries. The favourable preliminary results imply that this technique is a simple, safe and effective approach to coronary ostial repair in patients with type A aortic dissection or infective endocarditis.

## INTRODUCTION

Coronary artery (CA) ostial injury is usually seen in type A aortic dissection (TAAD) and infective endocarditis, which may lead to false lumen compression, intimal tear or disruption or circumferential ostial detachment with an inner cylinder intussusception [1]. Once the ostial injury progresses to coronary malperfusion, the mortality risk may be increased by 7-fold in patients with TAAD [2]. Currently available approaches to coronary ostial injury in such patients include direct repair with continuous suture, percutaneous coronary intervention and coronary artery bypass grafting (CABG). In our practice, we have developed a different technique for coronary ostial injury by using a ring-shaped bovine pericardial patch. The report seeks to describe this technique and evaluate the preliminary outcomes in 4 patients.

## PATIENTS AND METHODS

The Ethics Committee of Luoyang Central Hospital approved this retrospective study and waived the need for informed patient consent (No. LWLL22020708).

From August 2020 to January 2022, we treated 4 patients with coronary ostial injury by using a ring-shaped bovine pericardial patch. The mean age was 37 ± 11.4 years (range 26–53). The aetiology was TAAD in 3 patients and infective endocarditis in 1 patient. TAAD involved the left coronary ostium in 2 patients and the right coronary ostium in 1. Preoperatively, transthoracic echocardiogram and aortic computed tomographic angiogram (CTA) did not identify any injury to the coronary ostium or aortic regurgitation, nor did electrocardiograph (ECG) show any signs of myocardial ischaemia.

After the ascending aorta was opened, the coronary ostial intima was found to be split into long fissures located close to the bottom of the ostium in all patients. In the patient with infective endocarditis, the vegetation on the aortic valve invaded the intima below the left coronary ostium, which was ulcerated and dissected, leading to a concomitant intimal intussusception involving the proximal segment of the left main CA.

**Figure 1:**
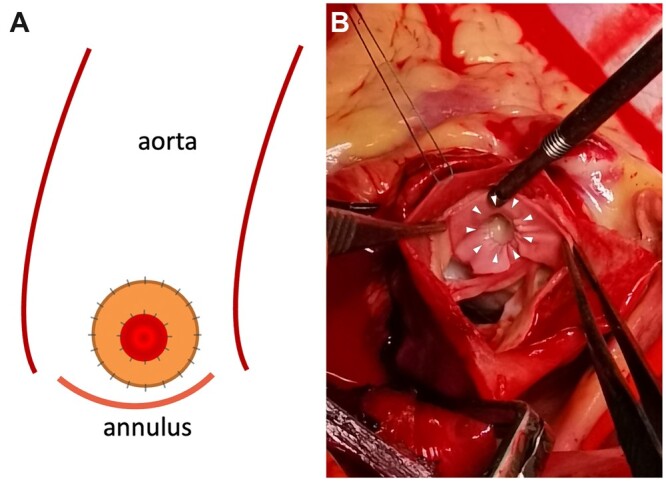
(**A**) Diagram of coronary ostial repair with a ring-shaped bovine pericardial patch. The red inner circle and yellow outer ring stand for the coronary ostium and the bovine pericardial patch, respectively. (**B**) Operative view of right coronary ostial repair with a ring-shaped bovine pericardial patch in a 35-year-old male with type A aortic dissection involving the right coronary ostia. The arrowheads point to the inner rim of the patch that was sutured in place to the right coronary ostium. (A color version of this figure appears in the online version of this article.)

A piece of bovine pericardium (Beijing Balance Medical, Beijing, China) was carefully trimmed into a ring-shape patch; the inner and outer diameters were ∼5 and 20 mm, respectively, so that the patch could cover the involved coronary sinus (Fig. 1A). The inner rim of the bovine pericardial patch was continuously sutured to the coronary ostium with 7–0 prolene. To avoid deterioration of ostial dissection and minimize the risk of postoperative coronary occlusion, 7–0 prolene suture was used in the first stitch, which was sewn obliquely on both sides of the ostial tear, completely covering the torn ostium with bovine pericardium (Fig. 1B). In the case of infective endocarditis, to address the inner cylinder intussusception of left main CA, the inner rim of the ring-shaped patch, coronary intima and aortic adventitia were continuously sutured with 6–0 prolene in a sandwich manner. Due to the fragility of tissue in acute aortic dissection and endocarditis, a full-thickness bite was taken to transverse the aortic wall to strengthen repair. To minimize the potential risk of creating a new tear within the artery while suturing the dissected coronary ostia, the needle and prolene should be handled very gently, with extreme care to avoid excessive force and tension on the suture and ostial intima. The outer rim of the bovine pericardial patch was continuously sewn to the aortic wall with 6–0 prolene suture. No pledgets were placed outside the aortic adventitia.

## RESULTS

The procedure was successful in all patients. Coronary ostial repair lasted for 10–15 min. The heart resumed beating automatically after the cross-clamp was removed. The primary operations were ascending aortic repair and total arch repair with a frozen elephant trunk for acute type A dissection, and aortic valve replacement with a mechanical prosthesis for infective endocarditis. Neither mortality nor morbidity occurred, and all patients were discharged in good condition.

**Figure 2:**
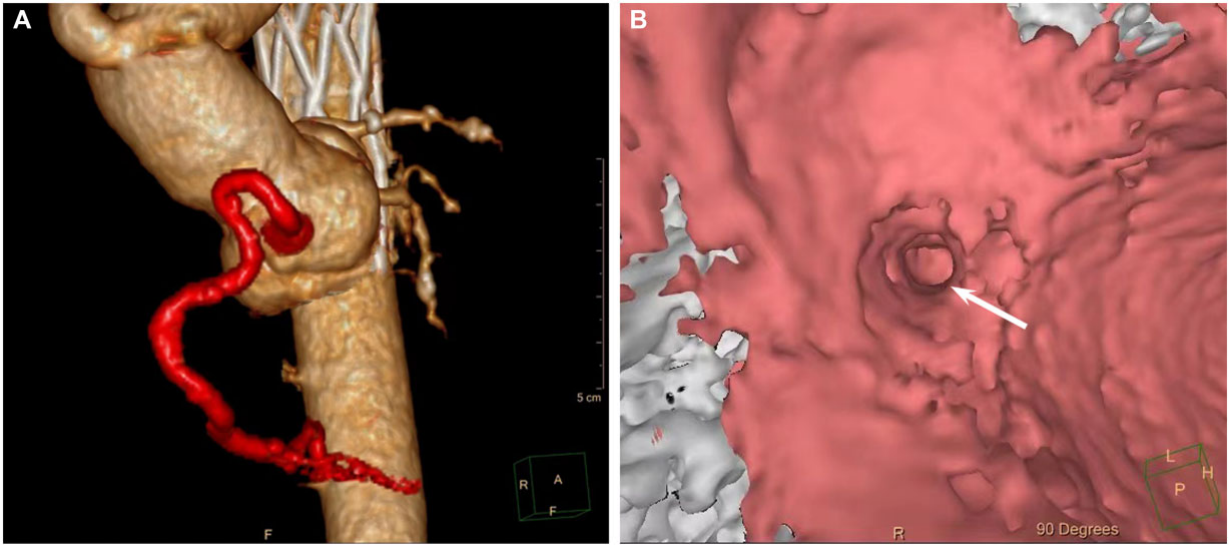
Follow-up coronary CT arteriogram of the same patient in Fig. 2. (**A**) Volume-rendered CT images showed a well visualized right coronary artery with patent lumen. (**B**) Endoscopic images of the aortic root showed a patent right coronary ostium (arrow).

Clinical and imaging follow-up was complete in 100% at a mean duration of 1.6 ± 0.7 years (range 0.5–1.9). All patients were doing well and without symptoms at the latest follow-up. ECG did not reveal any signs of myocardial ischaemia. In all patients, coronary computed tomographic angiogram showed a normal and patent coronary ostium, unobstructed distal blood flow to left CA, with no signs of coronary ostial stenosis or dissection (Fig. 2).

## DISCUSSION

Coronary malperfusion occurs in ∼7% of patients with acute TAAD and surgical strategies depend on the severity of the CA dissection. According to lesions of coronary malperfusion, Neri *et al.* [1] classified the CA dissection into 3 subtypes: ostial dissection, dissection with a coronary false channel and circumferential detachment with an inner cylinder intussusception. The latter 2 subtypes are usually managed with CABG or percutaneous coronary intervention [3], while the optimal approach to ostial dissection remains unclear, and composite root replacement is not reasonable in the absence of moderate or severe aortic regurgitation and may put patients at coagulation-related risks. Although direct suture with 7–0 prolene is widely used, the anastomosis is often under high tension and at risk of anastomotic breakage or leak. While supracoronary aortic replacement can resolve most dissections located at or close to the top of the ostium, it may not be effective in addressing dissections located at the left or right side and close to the bottom of the coronary ostia. Therefore, ring-shaped repair with bovine pericardial patch may be a good approach for the latter case. Compared to CABG and composite graft or valve-sparing root replacement (Bentall or David), our technique can reinforce the sinuses of Valsalva in an easier manner and may avoid the risk of bleeding and thromboembolic complications associated with anticoagulation therapy and minimize the risk of deterioration of coronary ostial injury. The preliminary results in our patients suggest that ostial repair with a ring-shaped bovine pericardial patch is a feasible approach to coronary ostial injury in TAAD or infective endocarditis.

Barbero *et al.* reported a somewhat similar technique for catheter-induced left main coronary dissection with subsequent retrograde progression into the ascending aortic wall. The left coronary ostium was repaired with an autologous ring-shaped aortic patch, which achieved unobstructed blood flow in left CA postoperatively [4]. The biggest advantage of an autologous aortic patch lies in its smooth surface that reduces the risk of thrombosis. It offers more strength in buttressing the coronary ostium and sinuses compared to direct suture repair, which effectively avoids progression of the ostial injury caused by blood flow and aggravation of myocardial ischaemia, thereby eliminating the need for CABG. In contrast, a ring-shaped bovine pericardial patch offers more advantages compared to an autologous aortic or pericardial patch. It is thinner and less likely to cause ostial and proximal coronary stenosis, especially when the intima of coronary ostium is involved in the repair. Bovine pericardial patch is pliable and strong and holds the stitches more reliably than an autologous aortic patch does, which is often brittle and oedematous in acute dissection or infective endocarditis. It is stronger than an autologous pericardial patch, which is thicker and takes longer time to suture. However, the bovine pericardial patch may calcify over time, which needs to be closely monitored.

Although the favourable results in our patients suggest that the ring-shaped bovine pericardium repair is safe and effective for coronary ostial injury in acute aortic dissection or infective endocarditis, further studies in more patients for longer duration are warranted to evaluate its long-term efficacy and durability.

**Conflict of interest:** none declared.

## Data Availability

The data underlying this article will be shared on reasonable request to the corresponding author.

## References

[1] Neri E , Toscano T, Papalia U, Frati G, Massetti M, Capannini G et al Proximal aortic dissection with coronary malperfusion: presentation, management, and outcome. J Thorac Cardiovasc Surg 2001;121:552–60. 11241091 10.1067/mtc.2001.112534

[2] Ma WG , Chen Y, Zhang W, Li Q, Li JR, Zheng J et al Extended repair for acute type A aortic dissection: long-term outcomes of the frozen elephant trunk technique beyond 10 years. J Cardiovasc Surg (Torino) 2020;61:292–300. 10.23736/S0021-9509.20.11293-X 32077675

[3] Tang YF , Zhang GX, Liao ZL, Han L, Xu ZY. Surgical treatment of coronary malperfusion with acute type A aortic dissection. Chin Med J (Engl) 2016;129:1000–2. 27064047 10.4103/0366-6999.179797 PMC4831516

[4] Barbero C , Di Rosa E, Devotini R, Attisani M, Rinaldi M. Left main coronary artery ostial repair with autologous ring-shaped aortic patch for iatrogenic aortic dissection. J Card Surg 2014;29:821–3. 25269622 10.1111/jocs.12446

